# Klotho’s impact on diabetic nephropathy and its emerging connection to diabetic retinopathy

**DOI:** 10.3389/fendo.2023.1180169

**Published:** 2023-04-18

**Authors:** Anqi Tang, Yu Zhang, Ling Wu, Yong Lin, Lizeyu Lv, Liangbin Zhao, Bojun Xu, Youqun Huang, Mingquan Li

**Affiliations:** ^1^ Department of Nephrology, Hospital of Chengdu University of Traditional Chinese Medicine, Sichuan, China; ^2^ Department of Nephrology, Shaanxi Provincial Hospital of Traditional Chinese Medicine, Xi’an, China

**Keywords:** klotho, diabetic nephropathy, type 2 diabetes mellitus, diabetic retinopathy, mechanism, drug, chronic kidney disease

## Abstract

Diabetic nephropathy (DN) is the leading cause of end-stage renal disease worldwide and is a significant burden on healthcare systems. α-klotho (klotho) is a protein known for its anti-aging properties and has been shown to delay the onset of age-related diseases. Soluble klotho is produced by cleavage of the full-length transmembrane protein by a disintegrin and metalloproteases, and it exerts various physiological effects by circulating throughout the body. In type 2 diabetes and its complications DN, a significant decrease in klotho expression has been observed. This reduction in klotho levels may indicate the progression of DN and suggest that klotho may be involved in multiple pathological mechanisms that contribute to the onset and development of DN. This article examines the potential of soluble klotho as a therapeutic agent for DN, with a focus on its ability to impact multiple pathways. These pathways include anti-inflammatory and oxidative stress, anti-fibrotic, endothelial protection, prevention of vascular calcification, regulation of metabolism, maintenance of calcium and phosphate homeostasis, and regulation of cell fate through modulation of autophagy, apoptosis, and pyroptosis pathways. Diabetic retinopathy shares similar pathological mechanisms with DN, and targeting klotho may offer new insights into the prevention and treatment of both conditions. Finally, this review assesses the potential of various drugs used in clinical practice to modulate klotho levels through different mechanisms and their potential to improve DN by impacting klotho levels.

## 1 Introduction

The incidence of Diabetic Nephropathy (DN), a microvascular complication caused by diabetes, has been on the rise globally and is currently the primary cause of end-stage renal disease (1). In most cases, the presence of persistent albuminuria in patients with type 2 diabetes mellitus (T2DM), along with concomitant retinopathy and the exclusion of other renal diseases, serves as the main diagnostic criteria for DN, as a renal biopsy can be traumatic for the patient. DN affects approximately 40% of patients with T2DM, and some studies indicate a percentage as high as 55% (2, 3). Patients with DN are at a significantly higher risk of experiencing cardiovascular events and all-cause mortality, requiring continuous renal replacement therapy when they progress to end-stage renal disease, which places a heavy burden on both society and families due to the scarcity of healthcare resources.

The pathogenesis of DN is multifactorial, involving various mechanisms such as activation of the renin-aldosterone system, glomerular hyperfiltration, altered renal hemodynamics, ischemia and hypoxia, oxidative stress, inflammation, and fibrosis (4). Several classes of drugs, including hypoglycemic, agents renin-angiotensin system (RAS) blockers, sodium-glucose cotransport protein-2 inhibitors (SGLT2i), and glucagon-like peptide-1 receptor (GLP-1R) agonists, have been shown to effectively manage diabetes and DN (5). However, the progression of DN can occur in patients who are transiently exposed to hyperglycemia, even when their glycemia is mostly controlled within the normal range by medication (6, 7). This indicates that the pathogenesis of DN is still not fully understood, and the available drugs cannot completely halt the persistence of the pathological state. Given the gravity of the impact of DN on patient prognosis and the healthcare system, it is imperative to pursue intensive research with a focus on developing drugs that can precisely intervene in the disease’s pathogenesis.

**Figure 1:**
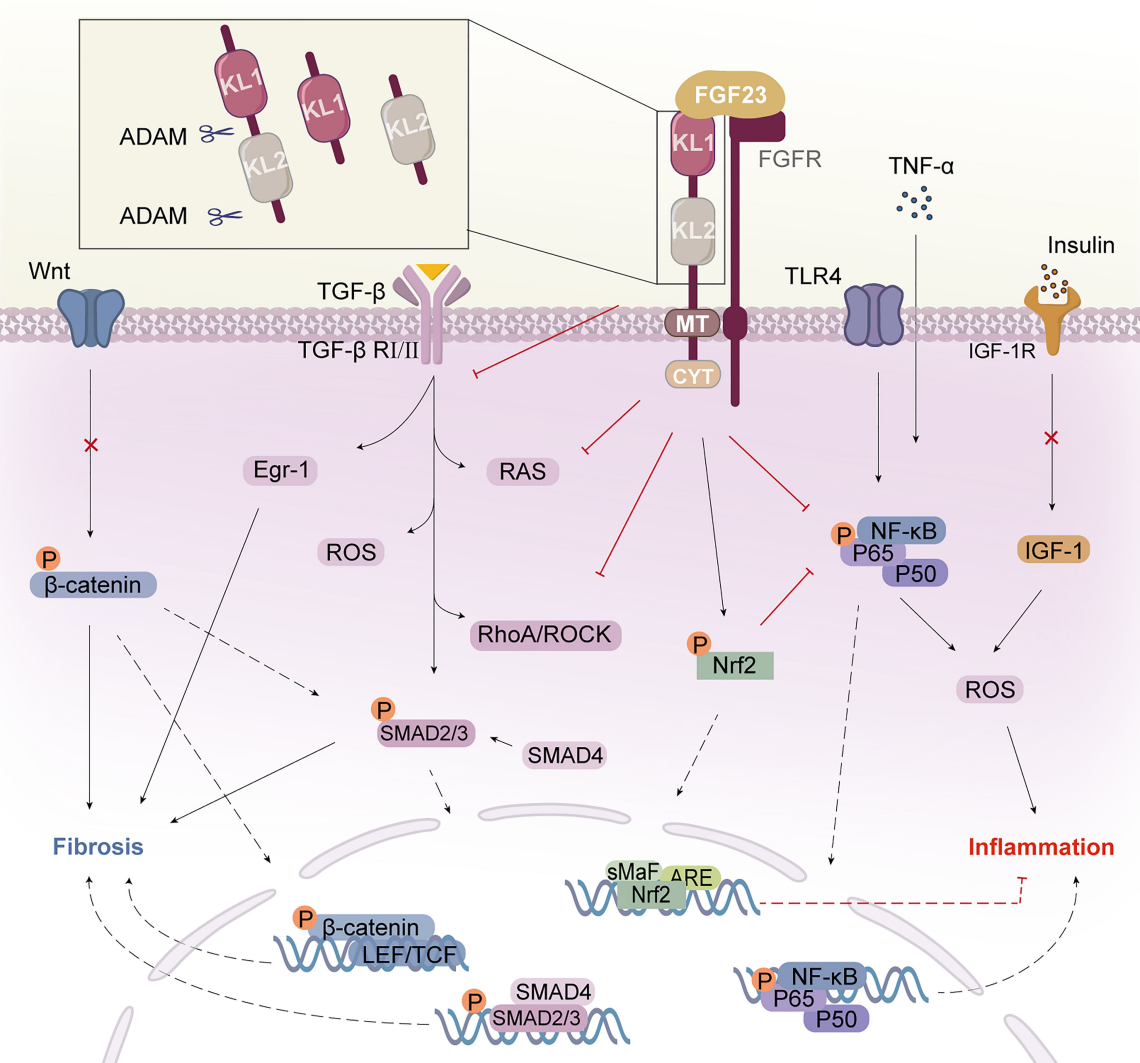
Klotho structure and its mechanism of action. Klotho consists of two extracellular structural domains (KL1 and KL2), a transmembrane segment (TM), and a short non-signaling cytoplasmic tail (CYT). Enzymes such as ADAM10 or ADAM17 can cleave Klotho to produce a soluble form called s-Klotho. FGF23 binds to the Klotho/FGFR1c receptor in the renal tubule to form the Klotho/FGFR/FGF23 signaling complex, which exerts multiple actions. Klotho binds to the TGF-β receptor, specifically the TβRII component, and inhibits TGF-β and its downstream pathways and molecular activation. Klotho inhibits the activation of the inflammatory NF-κB pathway and increases signaling in the Nrf2 pathway. Klotho block IGF-1 receptor signaling as well as the expression of target genes downstream of Wnt/β-catenin. Klotho inhibits inflammation and fibrosis in diabetic nephropathy through multiple pathways. KL, Klotho; FGF23, fibroblast growth factor 23; FGFR, FGF receptor; AMAD, a disintegrin and metalloproteases; TGF-β, transforming growth factor β; TβRII, TGF-β receptor type II; Egr-1, early growth response factor 1; RAS, agents renin-angiotensin system; ROS, reactive oxygen species; TLR4, toll-like receptor 4; Nrf2, nuclear factor-erythroid 2-related factor 2; NF-κB, nuclear factor κB; IGF-1, insulin-like growth factor 1; IGF-1R, IGF-1 receptor.

Klotho, first discovered in aging mice in 1997, has been linked to the control of illnesses associated with aging (8). Genetic defects in klotho have been found to cause vascular calcification, hyperphosphatemia, nephropathy, growth retardation, multi-organ atrophy, and fibrosis (8–10). Expression of klotho has been detected mainly in the kidney, brain, and pancreas, but also in human germ cells and sperm (11). The klotho family consists of three members: α-, β-, and γ-klotho (12). In this article, we will only focus on the molecular mechanisms of α-klotho in DN. Unless otherwise specified, the term “klotho” specifically refers to α-klotho. Three forms of klotho protein with different functions have been identified, including full-length transmembrane klotho (m-klotho), truncated soluble klotho (s-klotho), and secreted klotho, although no klotho receptor has been identified (13). M-klotho, which is the transmembrane form, binds to the fibroblast growth factor receptor (FGFR) and forms a ternary complex atomic structure with Fibroblast growth factor (FGF)23 and FGFR1c to confer stability (9, 14). It acts as a protein that primarily regulates renal phosphate excretion and aids in FGF23 signaling (15, 16). FGF23, secreted from bone, is known to reduce serum phosphate and 1,25-dihydroxy vitamin D levels through its action in the kidney (17). The extracellular structural domain of transmembrane klotho can be cleaved by a disintegrin and metalloproteinase (ADAM)-10 and ADAM-17, resulting in the formation of soluble klotho, which can be further cleaved to produce functionally independent Kl1 and Kl2 fragments and participate in the circulation (18) (**Figure 1**). Although klotho is predominantly expressed in the renal distal convoluted tubules, both tubular and glomerular compartments are protected by this circulating hormone (19, 20). This is because secreted klotho circulates in the blood and acts on tissues or cells that do not express klotho (9, 13). Furthermore, Secretory klotho exhibits potential sialidase activity, which modifies cell surface glycans. This feature may be related to klotho’s ability to regulate multiple ion channels, including transforming growth factor-β1 (TGF-β1), insulin-like growth factor-1 (IGF-1), Wnt, and growth factors such as insulin (21). Circulating levels of soluble klotho decrease with age and are reduced in pathological conditions such as DN, acute kidney injury, and chronic wasting disease (22, 23).

With further research, klotho levels may be linked to the development of various pathological mechanisms, such as metabolic disorders, oxidative stress, inflammation, and fibrosis. Despite extensive research on klotho, there is currently no comprehensive literature summarizing its specific mechanisms of action in DN. Therefore, in this paper, we provide the first analysis and description of the multiple ways in which klotho is involved in the development and progression of DN, based on existing studies. Importantly, targeting klotho may be a promising strategy for preventing and treating DR and DN, given their shared pathological mechanisms. In light of the high cost and long duration of drug development, we also compile a list of drugs commonly used in clinical practice, which may have potential in improving DN by modulating klotho levels.

## 2 Potential relationship between klotho and diabetes and DN

Klotho is involved in adipocyte maturation and systemic glucose metabolism and is closely related to the development of T2DM (24, 25). Klotho levels show a decreasing trend in both type 1 diabetes mellitus (T1DM) (26–28) and T2DM (29–31), and it is even depleted in the pancreas of diabetic patients (32). Surprisingly, it has been suggested that patients with poor glycemic control may have higher s-klotho levels, possibly because glycosuria increases the metabolic demands of the renal tubules, leading to increased expression and/or cleavage of klotho (33).

It should be noted that low levels of circulating klotho have a strong association with deteriorating renal function in patients with T2DM, with this trend being more pronounced in those receiving hemodialysis (33). Furthermore, studies have indicated that the expression of klotho in both plasma and urine is reduced in the early stages of DN (34–36) and that a further decrease may indicate the development of DN (37). In simpler terms, the levels of serum soluble klotho have a positive correlation with the estimated glomerular filtration rate (eGFR) and a negative correlation with urinary albumin, particularly in patients with diabetes (29, 30, 38–40). Meanwhile, one study mentioned that the ratio of klotho/Cr in random urine tends to decrease as eGFR decreases (41). Conversely, increasing klotho levels helped to delay the progression of diabetes and postpone the onset of diabetic complications (31, 32). All these results suggest that klotho plays a key role in the development of DN.

Nevertheless, it has been suggested that hyperglycemia does not directly impact klotho production in isolated human renal tubular epithelial cells (42). However, this conclusion may be premature as it fails to account for the effect of the entire renal system and its potential paracrine and endocrine regulators. There is evidence suggesting that the level of klotho is not related to kidney function, but is significantly associated with age (43). This may be attributed to the inconsistency of the primary disease and comorbidities in the study, which can contribute to a complex physiological and pathological process. In Addition, certain drugs, such as statins and angiotensin II receptor blockers, interfere with klotho expression, leading to inconsistent results (44–46). In summary, directing therapeutic efforts towards klotho presents a promising avenue for treating patients with DN and may also offer benefits for individuals with T2DM.

## 3 Inflammation and oxidative stress

Several inflammatory factors, such as monocyte chemoattractant protein-1 (MCP-1), tumor necrosis factor-α (TNF-α), interleukin-1β (IL-1β), as well as oxidative stress factors, such as reactive oxygen species (ROS) and nitric oxide (NO), are involved in inflammation-related kidney injury in DN (47). The relationship between inflammation and oxidative stress is complex and interrelated, with inflammation leading to increased ROS production and ROS exacerbating inflammation, resulting in more severe oxidative stress and inflammatory response in DN patients (48). It has been observed that the levels of klotho decrease with increasing levels of inflammation and oxidative stress in DN patients (35). Studies have shown that inflammatory factors, including tumor necrosis factor-α (TNF-α), and the uremic toxin indolyl sulfate, can increase ROS production and downregulate klotho expression through a nuclear factor-kappaB(NF-κB)-dependent mechanism (49, 50). In addition, epigenetic involvement in klotho gene expression, such as m6A modification of the methyltransferase complex, has been found to reduce klotho expression and exacerbate renal injury and inflammation in DN (51).

Increased levels of klotho help to control levels of inflammation and oxidative stress. Klotho attenuates the inflammatory response induced by hyperglycemia by directly or indirectly regulating NF-κB, thereby reducing renal damage (35, 52, 53). It can be argued that klotho is an anti-inflammatory modulator that negatively regulates NF-κB, and thus reduces the transactivation of pro-inflammatory genes, primarily through a mechanism involving phosphorylation of Ser (536) in the transactivating structural domain of RelA (53). Klotho also inhibits the translocation of NF-κB to the nucleus and attenuates the activity of the NF-κB pathway, thereby reducing the production of downstream inflammatory factors (54).

Klotho has been demonstrated to possess an inhibitory effect on toll-like receptor 4 (TLR4), resulting in the alleviation of inflammation and fibrosis in mesangial cells cultured in high glucose (52). Activation of TLR4 is also known to contribute to the transcription of pro-inflammatory genes, production of inflammatory factors, and ROS, promoting the development of systemic inflammation through paracrine and systemic effects. Subsequent investigations have demonstrated that klotho and TLR4 counteract each other *via* a protein hydrolysis process related to deglycosylation, whereby klotho mitigates the downstream inflammatory response associated with TLR4 and reverses acute kidney inflammation (55).

In early DN, IGF-1 activity is increased and interacts with the RAS to co-regulate renal hemodynamics (56, 57). Specifically, in renal tubular epithelial cells, IGF-1 induces elevated levels of ROS, NADPH oxidase activity, and fibronectin expression, which trigger renal inflammation (58). Conversely, klotho inhibits intracellular insulin/IGF-1 signaling and enhances tissue resistance to oxidative stress by suppressing tyrosine phosphorylation of insulin and IGF1 receptors (19, 57, 59). In light of this, Takenaka et al. proposed that klotho protein supplementation may have the potential to alleviate hypertension and albuminuria (57).

Furthermore, overexpression of klotho has been shown to significantly induce the signaling of nuclear factor erythroid 2-related factor 2 (Nrf2), a core transcription factor that regulates antioxidant responses. By regulating Nrf2 and cytoprotective antioxidant enzymes, klotho inhibits high glucose-induced oxidative stress and podocyte apoptosis, thereby ameliorating DN (60, 61). Consequently, klotho may have the immunotherapeutic potential for the treatment of diabetes and its associated nephropathy by controlling levels of inflammation and oxidative stress.

## 4 Fibrosis

A decrease in klotho levels can have a significant impact on endocrine signaling in the body, and a reduced abundance of klotho in the kidneys may directly lead to renal fibrosis and impaired renal function (62–64). Specifically, the klotho protein inhibits the binding of TGF-β1 to cell surface receptors, thereby inhibiting the TGF-β1-induced epithelial-mesenchymal transition (EMT) response, which is a crucial mechanism underlying renal fibrosis (65). In other words, low levels of klotho can activate the TGF-β1 signaling pathway, which contributes to diabetes-related renal fibrosis (66, 67). Notably, when klotho is deficient, a large increase in circulating levels of FGF23 can also target cell types that lack klotho, and this becomes the pathological basis for promoting fibrosis. However, it has been demonstrated that recombinant klotho protein or exogenous klotho supplementation can inhibit high glucose-induced TGF-β signaling, downregulate the expression of pro-fibrotic genes in renal mesenchymal fibroblasts, and ultimately reverse fibrotic lesions (57, 68, 69).

In-depth studies have shown that klotho down-regulates early growth response factor 1 (Egr-1) downstream through the inhibition of TGF-β1 signaling. This mechanism prevents the proliferation of glomerular mesangial cells (MC) and the overproduction of extracellular matrix (ECM) under hyperglycemic conditions (70). Egr-1 is expressed in renal tubular fibroblasts, glomerular mesangial cells, and several other cell types, making it a critical factor in the EMT of DN (71, 72). In particular, klotho has been found to directly target the Egr1/TLR4/mammalian target of rapamycin (mTOR) axis, which reduces inflammation and fibrosis in the glomerulus, suggesting that it has multi-targeted effects (73). Importantly, klotho has significant upstream regulatory effects on TGF-β1. For example, klotho can downregulate TGF-β1 and connective tissue growth factor expression by inhibiting RhoA/ROCK activity, thereby reducing tubulointerstitial fibrosis and proteinuria in DN (74). The results reported by Deng et al. are consistent with these findings and suggest that klotho may inhibit ROCK1 mRNA transcription and protein activity by suppressing NF-κB activity (75).

The Wnt/β-catenin proteins frequently interconnect with other signaling pathways, including TGF-β1, that jointly contribute to a complex mechanism promoting interstitial fibrosis in the kidney. The endogenous antagonist klotho blocks Wnt-triggered β-catenin protein activation and nuclear translocation, inhibiting podocyte dedifferentiation and mesenchymal transformation and ultimately restoring podocyte integrity (76, 77). In another example, klotho-derived peptide 6 has been shown to possess similar properties to klotho by inhibiting the expression of downstream target genes of the Wnt/β-catenin pathway. This effect has been shown to improve DN and reduce lesions of glomerulosclerosis and interstitial fibrosis (78). It is worth noting that klotho can further suppress renin-angiotensin-aldosterone system (RAAS) activation by inhibiting the Wnt/β-catenin pathway, as the overexpression of β-catenin or Wnt ligands can activate all RAS genes (79). This mechanism, which involves the presence of klotho and its ability to inhibit the Wnt/β-catenin pathway, has the potential to significantly delay renal fibrosis. Despite these observations, given the complexity of fibrosis and the involvement of multiple signaling pathways, it is possible that klotho exerts its antifibrotic effects through other undiscovered mechanisms. Therefore, ongoing research on the mechanisms of klotho action may reveal new therapeutic targets for the prevention and treatment of DN fibrosis.

In summary, in addition to its ability to modulate TGF-β1 signaling, klotho also demonstrates the ability to inhibit other fibrosis-inducing signaling pathways such as Wnt/β-catenin, Egr-1, and ROCK. These findings suggest that secreted klotho may have the potential to exert endogenous antifibrotic effects by concurrently suppressing multiple growth factors signaling pathways (65). Despite the current findings, the intricate nature of fibrosis and the numerous signaling pathways involved suggest that klotho may exert its anti-fibrotic effects through undiscovered mechanisms. Therefore, further research is necessary to elucidate klotho’s mechanisms of action, potentially uncovering new therapeutic targets for preventing and treating fibrosis in individuals with DN.

## 5 Prevention of endothelial dysfunction

**Figure 2:**
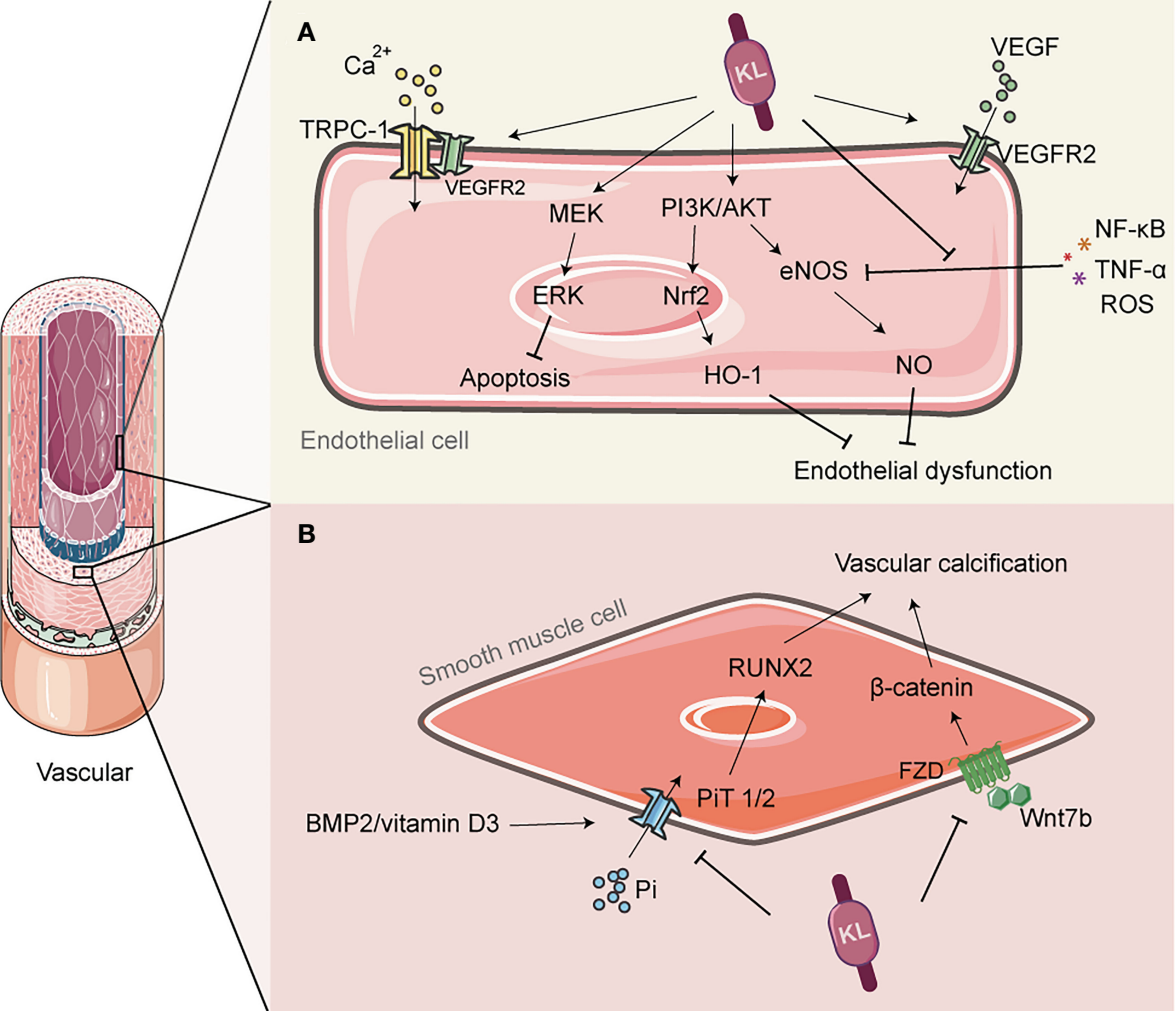
The role of Klotho in vascular protection. **(A)** Klotho can activate PI3K/AKT/Nrf2/HO-1 to enhance endothelial antioxidant defense; It can also activate PI3K/Akt/eNOS pathway, and alleviate the inhibition of eNOS phosphorylation by inflammatory factors such as TNF-α, thereby promoting NO production and preventing endothelial cell dysfunction. Klotho is involved in the transmission of VEGF signaling and regulates Ca2+ influx, which helps to maintain endothelial integrity. **(B)** Klotho inhibits phosphate uptake by vascular smooth muscle cells (VSMCs), leading to improved vascular calcification. It also inhibits the PiT2/RUNX2 signaling pathway, which improves extracellular matrix calcification. Additionally, Klotho inhibits the Wnt7b/β-catenin signaling pathway, which prevents VSMC calcification. NO, nitric oxide; eNOS, endothelial nitric oxide synthase; PI3K, Phosphoinositide 3-kinase; AKT, protein kinase B; Nrf2, Nuclear factor erythroid 2-related factor 2; HO-1, heme oxygenase-1; TRPC-1, transient-receptor potential canonical Ca(2+) channel 1; VEGF, vascular endothelial growth factor; PiT, Pi transporter; RUNX2, runt-associated transcription factor 2.

According to current understanding, glomerular endothelial cell (GEC) dysfunction is believed to be a key factor in the development of microangiopathy in DN, and it may also play a role in the failure of RAS inhibitors to effectively prevent the progression of DN (80). GEC dysfunction has several pathological manifestations, including endothelial cell apoptosis, impaired glomerular filtration barrier, and glycocalyx decomposition (81, 82), which may also contribute to the production of microalbuminuria (80). Previous research has shown that klotho gene expression is downregulated in several conditions of endothelial dysfunction associated with vascular disease, including DN (83). In addition, studies have shown that klotho protein or its metabolites promote endothelial NO production in small arteries through a humoral pathway, thereby maintaining normal endothelial function (83, 84) (**Figure 2A**). Further studies have shown that klotho plays an important role in the regulation of vascular function, vascular remodeling, and prevention of perivascular fibrosis through the upregulation of oxidative scavengers and activation of the phosphoinositide 3-kinase (PI3K)/protein kinase B (Akt)/nitric oxide synthase (eNOS) pathway, as well as stimulation of the MAPK/ERK kinase (MEK)/extracellular signal-regulated kinase (ERK) pathway to attenuate endothelial apoptosis and senescence (85–87).

In addition to regulating NO release from the endothelium, klotho may also play a role in the expression of vascular endothelial growth factor (VEGF), which is produced by podocytes and regulates the structure and function of neighboring endothelial cells in a paracrine manner, as well as regulating angiogenesis. It has been shown that klotho transmits VEGF signaling to transient-receptor potential canonical Ca(2+) channel 1 (TRPC-1), which regulates Ca2+ influx and thus helps to maintain endothelial integrity (88). Conversely, mice deficient in klotho exhibit increased hyperpermeability due to enhanced apoptosis and reduced surface expression of vascular endothelial (VE)-cadherin, which is accompanied by hyperactivation of Ca2+-dependent calpain/caspase-3 (88). However, the role of VEGF in the kidney is complex, and excess VEGF may be damaging to the kidney (89). At least one recent study has reported that klotho can inhibit VEGF secretion from the basal membrane by inhibiting phosphorylation of VEGF receptor 2, suggesting that klotho may have a bidirectional role in regulating the renal VEGF system (90). Furthermore, oxidative stress and inflammatory responses induced by high glucose are thought to be among the determinants of endothelial dysfunction (91). The anti-inflammatory and antioxidant effects of klotho are fully described in subsection 4 of this paper, but it is worth adding that klotho can also enhance the antioxidant defense of endothelial cells by activating PI3K/AKT/Nrf2/heme oxygenase-1 (HO-1) (92). In addition, klotho inhibits the expression of vascular cell adhesion molecule-1 (VCAM-1) and suppresses the inflammatory response and vascular damage caused by the adhesion of leukocytes to endothelial cells; at the same time, klotho also deregulates the inhibition of eNOS phosphorylation by TNF-α, maintains NO production and regulates endothelial inflammation (93). Overall, klotho may indirectly reduce endothelial dysfunction induced by inflammatory factors and uremic toxins through its anti-inflammatory and antioxidant effects (94).

## 6 Regulation of calcium and phosphate homeostasis

Calcium and phosphate are vital in the composition of biological substances, signal transduction, and other life processes. Multiple molecules are responsible for regulating their balance in the body, including parathyroid hormone (PTH), vitamin D, and klotho. In patients with T2DM as well as DN, high blood phosphorus levels are not only associated with cardiovascular mortality but also independently with renal disease progression (95, 96). As an important pathway for calcium and phosphorus excretion, increased urinary phosphorus excretion by the kidneys seems to guarantee a lower risk of chronic kidney disease (CKD) progression in T2DM patients (97). The mechanism by which klotho regulates calcium and phosphate homeostasis involves the binding of skeletal-produced FGF23 to the klotho/FGFR1c receptor in the renal tubules, which leads to increased phosphate excretion in the urine (98). Conversely, defects in klotho or FGF23 contribute to hyperphosphatemia (99). However, one study has reported that klotho can transport renal phosphate independently of FGF23 (100). Although the exact mechanism is not fully understood, it is clear that klotho plays a role in regulating the balance of calcium and phosphate in the body.

Calcification is a small vessel disease characterized by extensive vascular calcification as the main pathological feature, and diabetes is one of the main risk factors predisposing patients with chronic kidney disease to calcification reactions (101, 102). Downregulation of the klotho gene in chronic renal failure is thought to be associated with the development of vascular calcification (VC) (103) Specifically, defects in klotho lead to phosphate retention, and elevated blood phosphate concentrations trigger calcium phosphate precipitation, a product that is adsorbed by the serum protein fetuin-A and induces ectopic calcification (104, 105). In the early stages of renal disease, a reduction in the co-receptor klotho can lead to an increased risk of vascular calcification and vascular senescence. This risk is further amplified by a compensatory increase in F23 (10, 106, 107). However, it is unclear whether this effect is due to circulating klotho or localized klotho in the vascular system. Rukov et al. demonstrated that neither normal nor calcified vascular systems in chronic renal failure express klotho (108). However, this finding contradicts the findings of Donate-Correa et al., who reported that klotho is expressed in calcified human arteries (109, 110). All studies conducted thus far have confirmed the beneficial effects of klotho on vascular calcification. Klotho can improve vascular calcification by inhibiting phosphate uptake by vascular smooth muscle cells (VSMC) and increasing urinary phosphorus excretion (103, 111). Further studies have revealed that this process may also involve the regulation of the Wnt7b/β-catenin signaling pathway by klotho, which indirectly inhibits hyper phosphate-induced VSMC calcification (112) (**Figure 2B**). It is worth noting that even after excluding the effect of hyperphosphatemia-induced calcification, calcification of the extracellular matrix can still occur in arteries due to the activation of bone morphogenetic protein (BMP2)/vitamin D3- (Pi transporters 2, PiT2)/runt-associated transcription factor 2 (RUNX2) signaling. However, klotho has been found to inhibit the activation of this signaling pathway and attenuate the calcification response (113). Considering these reasons, the replacement of klotho may have therapeutic potential for vascular calcification in DN, and this strategy should not be underestimated.

## 7 Regulation of glucose metabolism

In diabetic rat kidneys, insulin-like growth factor 1 (IGF1) and insulin-like growth factor-1 receptor (IGF-1R) expression are significantly elevated, which can lead to lipid accumulation in rat renal mesangial cells and dysfunction (114, 115). However, overexpression of klotho in mice can bind to cell surface receptors and inhibit the intracellular transmission of insulin and IGF1 (19, 116). Specifically, klotho antagonizes IGF-1R and subsequently inhibits downstream PI3K/AKT/mTOR signaling, which contributes to increased peroxisome proliferator-activated receptor-α(PPAR-α)transcriptional activity of genes that regulate metabolism, improve insulin sensitivity, and maintain glucolipid homeostasis (117). While klotho overexpression has been shown to inhibit insulin signaling, it does not necessarily result in insulin resistance and can still effectively harness its anti-aging properties. On the contrary, a deficiency in klotho has been demonstrated to worsen insulin resistance and raise blood glucose levels (116, 117). This phenomenon may be attributed to the expression of klotho in pancreatic islets and insulinoma β-cells, which boosts insulin secretion by upregulating the plasma membrane level of transient receptor potential V2 (32, 118). Nevertheless, some studies have proposed an alternate view that klotho regulates metabolism and enhances whole-body energy expenditure by promoting glucose uptake and glycogen synthesis, and by enhancing insulin sensitivity through unknown mechanisms (116).

The discovery of klotho in the cerebral choroid plexus revealed that its functions are not limited to regulating glucose metabolism in the peripheral system. According to a study, central klotho does not have any impact on fasting insulin or glucose levels. Instead, its influence on insulin secretion may depend on glucose levels. Furthermore, the study suggests that this regulatory effect remains unaffected by any changes in body weight (119). However, recent studies have challenged previous findings and suggest that impaired central α-klotho function may play a role in abnormal glucose metabolism and development of obesity (120). It is worth noting that abnormalities in glucose metabolism can also impact klotho expression. For instance, s-klotho concentrations are significantly lower in children with T1DM compared to healthy children when controlling for related diseases such as aging (28). Despite controversy, this suggests that klotho may exert its anti-diabetic and anti-obesity effects through multiple pathways. In summary, the bioavailability of klotho may directly impact glucose metabolism, which in turn affects the development and progression of T2DM and DN. Further studies are required to assess potential adverse effects associated with the use of this soluble hormone, and to monitor whether the body becomes dependent on this exogenous hormone after prolonged use.

## 8 Regulation of cell death

**Figure 3:**
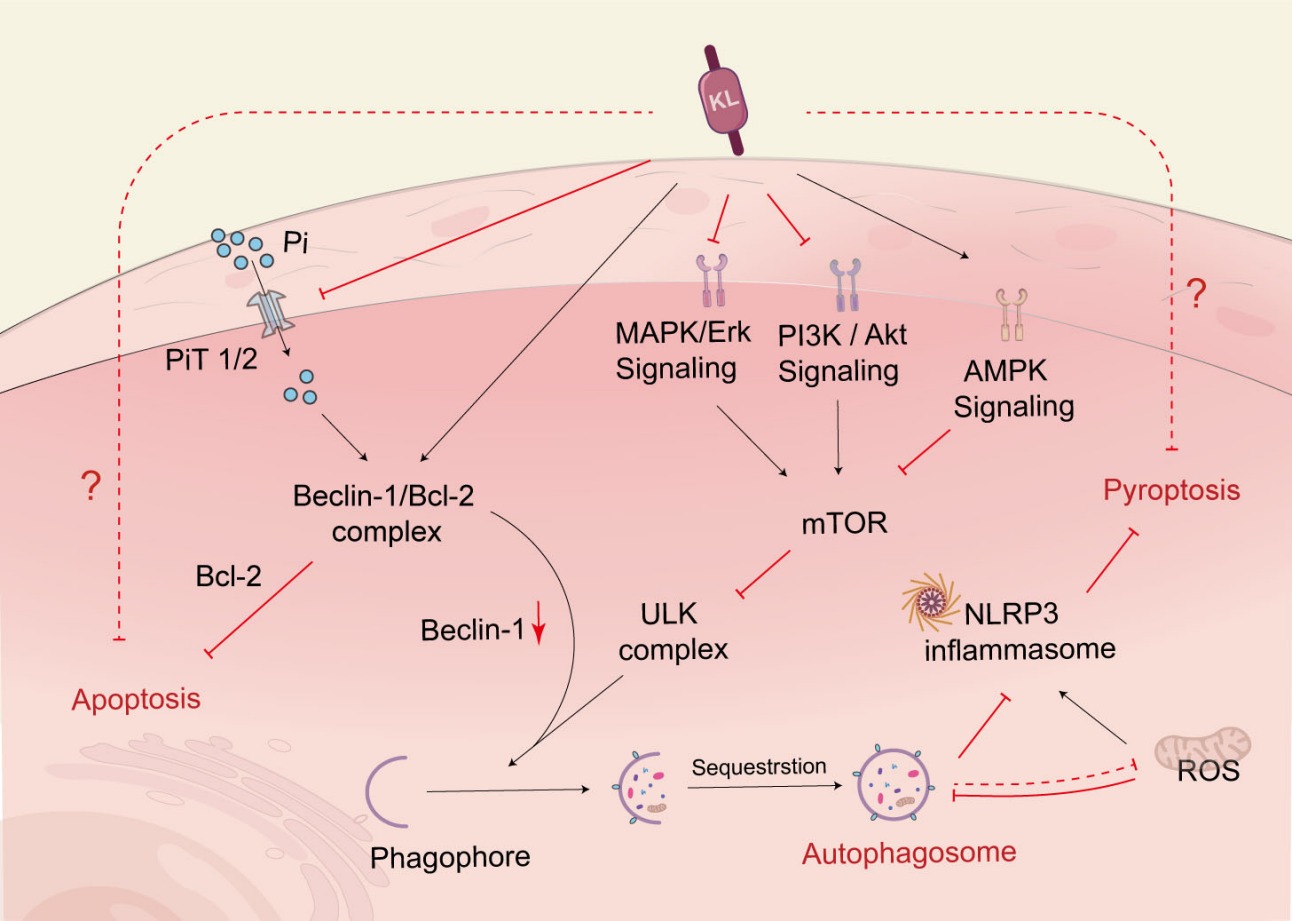
Klotho regulates cell death. Klotho regulates autophagic flux through PI3K/AKT/mTOR, MAPK, and AMPK pathways. Klotho can increase the binding of Beclin-1 to Bcl-2, reduce Beclin-1’s interaction with other autophagy-related proteins, and thereby regulate autophagy. Additionally, Klotho indirectly upregulates autophagy by inhibiting the activity of NLRP3 inflammatory vesicles, which helps prevent renal tubular cell death. Klotho also indirectly regulates autophagy by controlling the excretion of Pi. PI3K, phosphoinositide 3-kinase; AKT, protein kinase B; mTOR, mammalian target of rapamycin; MAPK, mitogen-activated protein kinase; AMPK, adenosine monophosphate-activated protein kinase; NLRP3, nucleotide-binding oligomerization domain-like pyrin domain-containing protein 3; PiT, phosphate transporter.

Autophagy is a lysosomal degradation pathway that aids cells in adapting to or counteracting stress responses, primarily by breaking down and recycling cytoplasmic components. Nutrient-sensing pathways, such as the IGF-1 signaling pathway, the mammalian target of rapamycin (mTOR) signaling pathway, and the adenylate-activated protein kinase (AMPK) pathway, regulate autophagy, with the target of mammalian rapamycin complex 1 (mTORC1) being the primary negative regulator of autophagy. A significant amount of evidence supports a regulatory role for autophagy in DN. For instance, cells exposed to high glucose levels for a prolonged period experience inhibited autophagy, which accelerates the development of DN (121). However, klotho can upregulate autophagy and mitigate renal cell injury and subsequent renal fibrosis (122). More specifically, klotho regulates the upregulation of autophagic flux by inhibiting the AKT/mTOR pathway or the IGF-1-mediated PI3K/Akt/mTOR pathway to improve diabetes and kidney injury (123, 124) (**Figure 3**). In parallel, the AMPK and MAPK pathways are also targets of klotho’s intervention to inhibit hyperglycemic damage to renal tubules through improved autophagy (121, 125).In addition to the autophagic pathways mentioned above, klotho also regulates Beclin-1, a crucial autophagy regulator, and may modulate autophagy levels by affecting the relative levels of the Beclin-1/Bcl-2 protein complex (124, 126). Specifically, klotho increases the binding of Beclin-1 to Bcl-2 and decreases the interactions of Beclin-1 with other autophagy-related proteins, thereby inhibiting autophagic activity in DN (127). It is important to note that the autophagic process is not only regulated by klotho but also influenced by Pi, which promotes the binding of Beclin-1 to its negative regulator BCL-2, impairing autophagic flux (128). Therefore, klotho can indirectly regulate autophagic flux by regulating Pi excretion as well.

In addition to its effects on the autophagic pathway, klotho is also known to rescue hyperglycemia-mediated apoptosis of podocytes, glomerular endothelial cells, and renal tubular epithelial cells (51, 60, 129). Interestingly, klotho can even inhibit apoptosis or necrosis caused by ischemia-reperfusion injury and nephrotoxicity (130, 131). It is widely recognized that apoptosis is primarily mediated by the mitochondrial pathway, the death receptor pathway, and endoplasmic reticulum stress. However, studies investigating the mechanisms by which klotho intervenes in apoptosis have only begun to scratch the surface of this complex process. As a molecular chaperone of klotho, HSP expression increases in response to various stresses and thus functions as a cytoprotective agent. Studies have shown that klotho reduces apoptosis *via* HSP-70 in experimental ischemic acute kidney injury, and the mechanism may involve HSP70 relying on the immunoprecipitated anti-apoptotic proteins Bcl-2 and Bcl-xl to provide protection to cells (132, 133). Recent research has also shown that HSP70 promotes renal cell survival by inhibiting nucleophosmin (NPM) phosphorylation and reducing its accumulation in the cytoplasm (134).

Pyroptosis is a type of programmed cell death that is characterized by cell lysis and is triggered by inflammatory vesicles. The canonical pathway for inducing pyroptosis is through activation of the nucleotide-binding oligomerization domain-like pyrin domain-containing protein 3 (NLRP3) inflammasome-mediated caspase-1. It is important to note that both pyroptosis and necroptosis pathways essentially result in inflammatory lytic cell death (135). Klotho was found to inhibit the Pyroptosis of renal tubular cells through the inhibition of NLRP3 inflammatory vesicles. The study also suggested that klotho might indirectly suppress the activity of NLRP3 inflammatory vesicles and the maturation of their downstream pathway molecules by upregulating autophagy (123).

In fact, autophagy, apoptosis, and pyroptosis are all part of programmed cell death. On the one hand, autophagy inhibits apoptosis as a pathway for cell survival, while on the other hand, autophagy itself or autophagy and apoptosis acting together can induce cell death. Apoptosis can be converted to pyroptosis *via* the cysteine-3-GSDME (caspase-3) signaling pathway (136), and autophagy in turn can inhibit pyroptosis by reducing mitochondrial ROS to suppress NLRP3 inflammatory vesicle activation (123). Therefore, these three mechanisms can act as both allies and adversaries, and the dominant mechanism is determined by the cellular environment in which the cell exists. Klotho is closely associated with cell fate and can exert its influence on the regression of DN by interfering with all three signaling pathways. However, the specific mechanisms through which it regulates cell death in the context of DN remain to be investigated in depth.

## 9 Emerging connection between klotho and diabetic retinopathy

**Figure 4:**
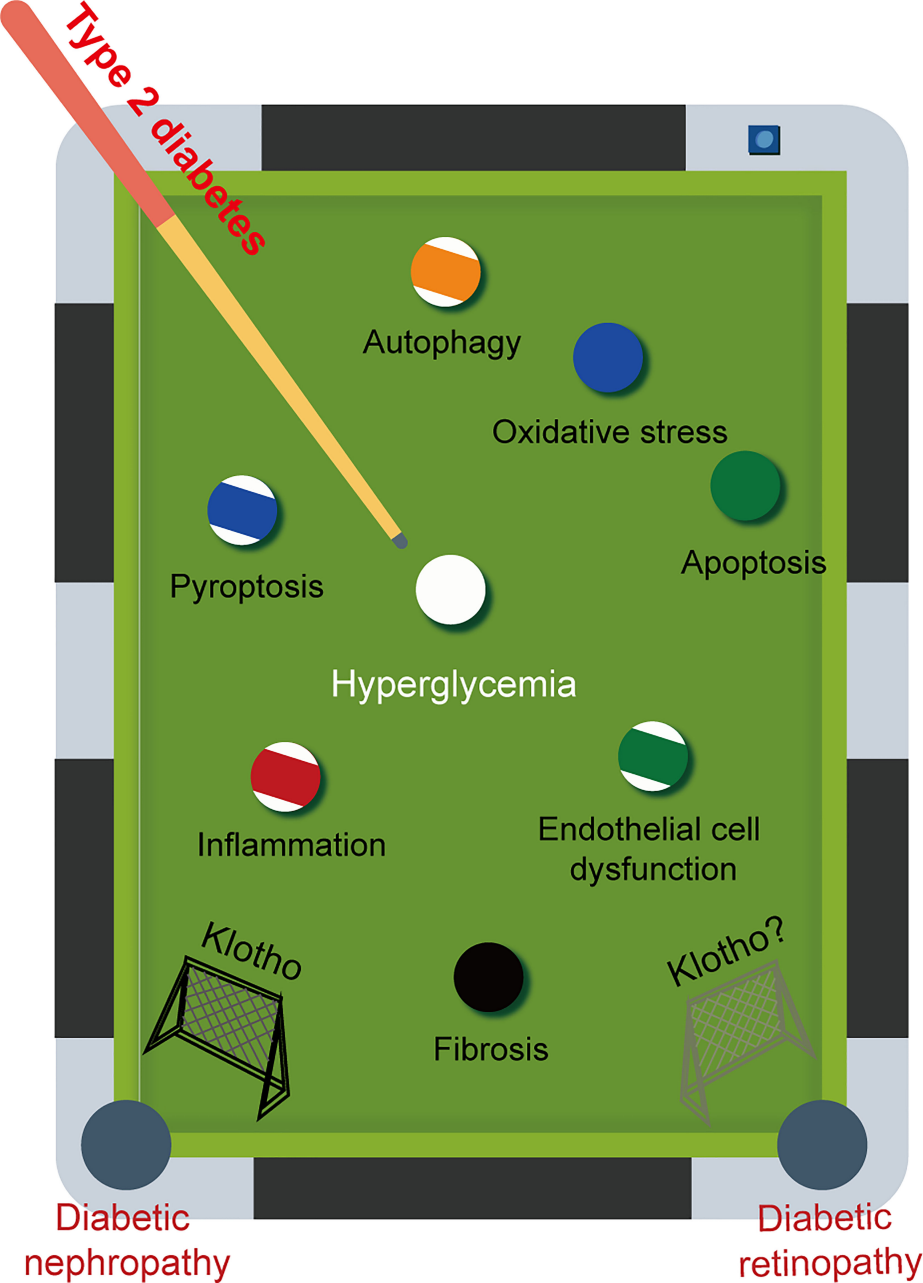
The game between pathogenic factors and Klotho. Diabetic retinopathy (DR) and diabetic nephropathy (DN) are two major microvascular complications that commonly occur in late-stage diabetes. These complications share common pathogenic mechanisms. Given the multiple beneficial effects of Klotho on DN, it is reasonable to speculate that Klotho may also have a positive effect on DR.

Klotho is found in every nuclear layer of the retina and is strongly linked to retinal function. For example, in klotho knockout mice, the amplitude of a- and b-waves is reduced, retinal signaling is weakened, and synaptic function is altered. Although there is no evidence of retinal degeneration, there is a definite tendency toward faster retinal aging (137). However, only a small number of therapeutic approaches targeting klotho have been explored for the treatment of DR. Late-stage diabetes can result in microvascular complications such as DR and DN. It is important to recognise that these complications share common pathogenic mechanisms and are often predictive of each other. Consistent with our expectation that decreased levels of klotho are also found in DR, and low levels of klotho are strongly associated with a high risk of progression to DR (138). Given the various advantageous effects of klotho on DN, it is speculated that klotho may also have a positive impact on DR (**Figure 4**).

Several studies revealed that decreased levels of α-klotho in the lens of diabetic rats lead to a reduction in Nrf2-mediated antioxidant defense, thereby contributing to increased NF-κB-mediated inflammatory responses. On the contrary, when klotho was supplemented, it resulted in elevated levels of antioxidants such as superoxide dismutase, glutathione peroxidase, glutathione, and other oxidants, thus effectively preventing oxidative stress and inflammation in the lens of diabetic rats (139, 140). The retinal pigment epithelium (RPE) is a polarized layer of cells containing pigment that is essential for normal visual function in the retina. The protein klotho plays a crucial role in maintaining the viability of the RPE by promoting mitochondrial biogenesis and scavenging ROS (141). In addition to its early effects on retinal function, klotho can also impact the late-stage morphology of the retina in DR. Specifically, klotho can indirectly inhibit secretion of VEGF from RPE cells by inhibiting insulin-like growth factor-3 (IGF-3) signaling and phosphorylation of VEGF receptor 4 (90). This function ultimately prevents neovascularization and helps to maintain the normal morphology of the choroidal layer, which is composed of blood vessels.

It is worth mentioning that klotho can exert a protective effect against DR by inhibiting retinal endothelial cell apoptosis. It achieves this by affecting the Bcl-2/Bax ratio and activating the PI3k/Akt signaling pathway (142). Indeed, autophagy, another form of programmed death, has an equally important role in the maintenance of retinal homeostasis. The relationship between apoptosis and autophagy is intricate. Since klotho can affect apoptosis, could it also exert a positive effect on DR by regulating autophagy? In addition, microvascular endothelial cells are considered to be the main target of hyperglycemic injury and diabetic endothelial dysfunction is a major cause of DR and DN development (82). However, there is still a lack of studies on the effects of klotho on microvascular endothelial cells in DR. In general, DR is the primary cause of blindness in adults with T2DM. But the specific mechanisms that contribute to this remain unknown. Given that klotho has demonstrated the ability to intervene in DN through various mechanisms and that there are similarities in the pathological mechanisms between DR and DN, targeting klotho as a therapeutic option may have the potential to intervene in DR.

## 10 Re-evaluation of commonly used clinical drugs

Targeting klotho may offer therapeutic benefits for DN through various pathways. However, the development of new drugs can be a very expensive and time-consuming process for a variety of reasons, including failure, poor safety profile, and limited efficacy. We, therefore, look at existing or commonly used clinical agents to explore whether they can have a positive impact on DN *via* klotho.

### 10.1 Anti-diabetic drugs

According to reports, metformin, which is the most commonly used antidiabetic drug, can increase klotho levels (143, 144). In addition, entertain-based therapies such as glucagon-like peptide-1 receptor agonist (GLP-1RA) and dipeptidyl peptidase-4 (DPP-4) inhibitors have also been found to increase klotho levels (145–147). However, it remains unclear whether these hypoglycemic agents directly increase klotho levels, modulates the inhibition of klotho by controlling blood glucose levels or indirectly increase klotho production by protecting relevant cells. It is certain that restoring normal blood glucose through insulin supplementation alone can also reverse lower klotho expression levels (148). Therefore, strict glycemic control is an effective approach to prevent the suppression of the physiological function of klotho.

Thiazolidinediones (TZDs) are agonists of peroxisome proliferator-activated receptor γ (PPAR-γ) and have been reported to induce klotho gene expression in a time- and dose-dependent manner in various renal epithelial cells, which may contribute to improving the symptoms and pathology of DN (149–151). That being said, the use of TZDs is limited due to their side effects, such as cardiovascular complications, fluid retention, and obesity, which have severely limited their use in clinical settings (152). New PPAR γ agonists with genetic selectivity have emerged as the next generation of anti-diabetic drugs, and they have shown fewer adverse effects than TZDs, which is encouraging (153). Nevertheless, further studies are needed to determine whether the effect of the new PPAR γ agonists on klotho has changed. The effects of various oral hypoglycemic agents (glimepiride, metformin, and linagliptin) on serum klotho levels are being compared in a trial registered with the Clinical Trials Registry (NCT03585777).

### 10.2 Sodium-glucose co-transporter inhibitors

Sodium-glucose co-transporter 2 (SGLT-2) inhibitors are widely known for their cardiorenal protective effects. Studies have shown that SGLT-2 inhibitors can restore klotho levels and improve diabetes and its associated cardiorenal complications (154–156). Specifically, SGLT-2 inhibitors prevent the downregulation of klotho by reducing the effects of glucotoxicity and inflammation on renal cells (157), which may contribute to the renal protection provided by SGLT-2 inhibitors. Recent studies have suggested that SGLT-2 inhibitors may not have an effect on klotho or FGF-23 levels, and no changes in serum calcium or phosphate levels were observed. However, this single-center study had a small number of subjects, and further trials are needed to confirm these findings (158). The effects of Empagliflozine (NCT02918591) and Dapagliflozin (NCT05033054, NCT05359263) on soluble klotho are currently being investigated.

### 10.3 Renin-angiotensin system blockers

RAS inhibitors can provide cardiorenal benefits that are independent of blood pressure through the upregulation of klotho expression (45, 159, 160). In turn, klotho can block RAS activation triggered by angiotensinogen and renin, resulting in a significant renoprotective effect (161). Additionally, Angiotensin Converting Enzyme-2, a component of the unconventional RAS, is strongly associated with the restoration of endogenous klotho levels (162). However, although some studies have shown that dual blockade therapy for diabetic nephropathy RAAS is superior to monotherapy, the relationship between this benefit and klotho is unclear (163).

### 10.4 Statin drugs

According to a study, statins were found to have vasoprotective effects without significant impacts on blood pressure or lipid levels (44). This suggests that statins may have vasoprotective effects that are independent of lipid metabolism. Further research indicates that atorvastatin or pitavastatin completely prevented the reduction of klotho expression in endothelial cells (44, 164, 165), indicating that the positive effects of these drugs on the kidney and blood vessels may be partially due to their ability to regulate klotho (160, 166). However, it is still unclear what specific role the antioxidant effects of statins play in influencing changes in klotho levels (165, 167).

### 10.5 Vitamin D

Vitamin D supplementation can normalize klotho levels in children with mild to moderate CKD, but in children with advanced CKD, the kidney’s ability to synthesize klotho has been reduced. Therefore, vitamin D supplementation may primarily promote increased levels of FGF23 (168). Moreover, aside from its ability to enhance endothelial cell adhesion, vitamin D also helps maintain endothelial integrity and functional stability, partially by increasing local klotho levels (169, 170). Although the source of increased klotho concentrations is uncertain, vitamin D still holds promise as a potential therapeutic approach for managing calcium and phosphorus metabolism and endothelial dysfunction in CKD patients (107, 169). A trial of oral vitamin D (Cholecalciferol) affecting plasma klotho levels has been completed but the results have not been published (NCT01524874). It is noteworthy that non-selective vitamin D receptor activators (VDRAs), such as calcitriol, have been linked to elevated levels of blood calcium and phosphate. In contrast, selective VDRAs prevent the adverse effects of increased blood calcium and serum phosphate levels, making them a more viable option in the future (171).

### 10.6 Mammalian target of rapamycin inhibitor

As commonly used autophagy inducers, mTOR inhibitors, such as rapamycin, have proven effective in treating DN as well as immunosuppression after organ transplantation (172–174). Some studies have shown that mTOR inhibitors can increase klotho expression in transplant recipients, although the effect of the transplanted kidney on klotho expression levels cannot be ruled out (175). In addition to this, mTOR inhibitors have indeed been shown to be effective agents in enhancing klotho levels (176, 177). However, these drugs suppress normal immune function and tend to cause abnormal increases in blood glucose, so their use in clinical practice is still limited.

### 10.7 Pentoxifylline

Pentoxifylline (PTX) is primarily utilized for the management of ischaemic peripheral vascular disease and plays a crucial role in the treatment of diabetic-related vascular disease and degenerative vascular complications (178, 179). PTX has been found to induce a dose-dependent increase in klotho protein and mRNA expression, with a concomitant increase in klotho levels detected in serum and urine. Furthermore, urinary klotho levels were found to be higher in stage 3 CKD patients than in those with more advanced CKD, suggesting a direct correlation between changes in klotho levels and eGFR (180). Given the enormous burden of diabetes and its complications, PTX should not be disregarded as a potential and reasonably accessible therapeutic alternative for DN, even in the absence of definitive data on renal outcomes.

### 10.8 Phytotherapeutics

Natural compounds derived from traditional medicines, such as resveratrol, cordycepin, astaxanthin, ligustilide, and ginseng, have been found to regulate klotho expression. They achieve this effect either by directly regulating the klotho gene or indirectly by regulating downstream molecules. These compounds are believed to have potential anti-aging (181–184), anti-oxidative stress (185, 186), fibrosis-ameliorating (187), cardiovascular calcification-improving (188), and organ-protective effects (189), either individually or simultaneously, through this mechanism (**Table 1**). Interestingly, research shows that Tetrahydroxystilbene glucoside, a major component of Polygonatum multiflorum, can prolong the lifespan of mice by modulating neuronal insulin signaling (190). In addition, baicalin and curcumin have been found to regulate endogenous klotho expression by modulating klotho promoter methylation, which can alleviate diabetes or cyclosporin A-induced kidney injury in mice (191, 192). Based on current evidence, it appears that both klotho and compounds targeting klotho have the potential to affect a variety of bodily systems, including the nervous, cardiovascular, and urinary systems. While traditional Chinese medical theory can guide the application of natural compounds in the human body, it remains unclear whether these compounds can effectively modulate klotho levels in humans. Therefore, further research is necessary to fully assess the potential of phytotherapeutics in targeting klotho.

**Table 1:** Drugs already in clinical use targeting klotho.

Drugs	Targets	References
Biguanide		
Metformin	klotho protein**↑**	(143, 144)
Dipeptidyl peptidase-4 (DPP-4)		
Linagliptin	regulate FGF-23/klotho expression	(145)
Vildagliptin	klotho protein**↑**	(146)
Sitagliptin	klotho protein**↑**	(147)
Sodium-glucose co-transporter 2 (SGLT-2) inhibitors		
Empagliflozine	increases or preserve klotho expression	(155, 156)
Canagliflozin	klotho protein**↑**, klotho mRNA**↑**	(156)
Dapagliflozin	klotho protein**↑**, klotho mRNA**↑**	(156)
Thiazolidinediones (TZDs)		
Troglitazone	activates PPAR-γ, klotho protein**↑**, klotho mRNA**↑**	(149)
Ciglitazone	activates PPAR-γ, klotho protein**↑**, klotho mRNA**↑**	(149)
Pioglitazone	klotho protein**↑**, klotho mRNA**↑**	(150)
Rosiglitazone	activates PPAR-γ, klotho protein**↑**, klotho mRNA**↑**	(151)
Renin-angiotensin-aldosterone inhibitors		
Losartan	klotho protein**↑**, klotho mRNA**↑**, preserve klotho expression	(45, 150)
Valsartan	klotho protein**↑**	(159, 160)
Statins		
Atorvastatin	preserve klotho expression	(44)
Pitavastatin	klotho protein**↑**, klotho mRNA**↑**, preserve klotho expression	(44, 164)
Simvastatin	klotho protein**↑**, klotho mRNA**↑**	(166)
Fluvastatin	klotho mRNA**↑**	(160)
Vitamin D Supplements		
Vitamin D	klotho protein**↑**	(168)
Calcitriol	klotho protein**↑**	(170)
Paricalcitol	klotho protein**↑**	(169)
mTOR inhibitor		
Rapamycin	klotho protein**↑**, klotho mRNA**↑**	(176, 177)
Nonspecific phosphodiesterase inhibitor		
Pentoxifylline	klotho protein**↑**, preserve klotho expression	(180)
Phytotherapeutics		
Cordycepin	klotho protein**↑**	(181)
Resveratrol	klotho protein**↑**, klotho mRNA**↑**, mediated expression of klotho *via* SIRT1	(182, 185, 188)
Astaxanthin	modulate klotho protein expression	(184)
Ligustilide	increases cleavage of full-length klotho by α-secretase	(183)
Ginseng	klotho protein**↑**, klotho mRNA**↑**	(186, 187)
Tetrahydroxystilbene Glucoside	klotho protein**↑**	(190)
Baicalin	regulates methylation of the klotho promoter	(191)
Curcumin	inhibits CpG hypermethylation of the klotho promoter	(192)
Dendrobium nobile Lindl. alkaloid	klotho protein**↑**	(144)

FGF-23, Fibroblast growth factor 23; peroxisome proliferator-activated receptor γ, PPAR-γ; Mammalian target of rapamycin, mTOR; silent information regulator factor 2-related enzyme 1, SIRT1.

## 11 Conclusion

Klotho plays a protective role in various cell types in DN, including renal tubular epithelial cells (72), renal glomerular and vascular endothelial cells (79, 86), mesenchymal fibroblasts (68, 69), and glomerular mesangial cells (70). Current evidence suggests that klotho can ameliorate DN through various mechanisms, including anti-fibrosis, anti-inflammatory and oxidative stress, vascular protection, metabolic modulation, anti-apoptosis, and regulation of autophagy. However, further research is needed to identify the primary targets of klotho’s action among these various pathways.

In recent years, sufficient attention has been paid to the study of the physiological role of klotho and related molecular mechanisms. Despite this, several questions surrounding its effects remain unresolved. For instance, a study found that serum glucose in the klotho overexpression group was significantly different from the control group 12 weeks after establishing a diabetic mouse model (193). However, it is still unclear whether klotho has the potential to impact serum glucose levels directly. In addition to blocking klotho downregulation or replacing deficient klotho, targeting microRNAs have been identified as an effective approach to modulating klotho levels (194, 195). Although, studies in this area are still in the preclinical stage. Furthermore, klotho has the potential to serve as a promising biomarker for the prediction of aging, endocrine or renal diseases. However, there is a need to refine its measurement techniques and diagnostic criteria.

Targeting klotho with approved drugs may be an effective approach to intervening in DN. Therefore, we have compiled several drugs in clinical use that modulate klotho levels through different mechanisms. According to current research, many drugs have been found to increase klotho concentrations, and klotho supplementation has not shown any signs of causing toxic side effects. While several clinical trials have been conducted to evaluate the efficacy of klotho in treating DN, further research is needed to determine the safety of long-term, high-dose administration of recombinant klotho or klotho supplements. Aside from assessing efficacy and safety, more research is necessary to examine the quantitative impact of these drugs in treating DN. Finally, although much of the fundamental research on klotho is based on animal models, further data from assays in healthy humans or patients with DN is required to refine our understanding.

## Author contributions

This research was conducted in collaboration with all authors. AT, and YZ were responsible for manuscript writing. LW, YL, LL, and LZ contributed to the manuscript revision. BX, YH, and ML contributed to the final approval. All authors contributed to the article and approved the submitted version.
